# Correlation of Hepatic Venous Pressure Gradient Level With Clinical and Endoscopic Parameters in Decompensated Chronic Liver Disease

**DOI:** 10.7759/cureus.51154

**Published:** 2023-12-27

**Authors:** Amol Sathawane, Harshal Khobragade, Sandip Pal

**Affiliations:** 1 Gastroenterology, Rabindranath Tagore International Institute of Cardiac Sciences, Kolkata, IND; 2 Medicine, Rabindranath Tagore International Institute of Cardiac Sciences, Kolkata, IND

**Keywords:** model for end-stage liver disease (meld), hepatitis b, chronic liver disease, alcohol-related liver disease, hepatic venous pressure

## Abstract

Background: The amount of liver fibrosis usually correlates with portal pressure, which is measured as the hepatic venous pressure gradient (HVPG). The fact that portal pressure significantly decreases after treatment may increase cirrhotic patients' long-term survival suggests that measuring HVPG may offer specific information for outcome prediction. The study thus seeks to determine the relationship between the level of the HVPG and endoscopic and clinical parameters in decompensated chronic liver disease (CLD).

Methodology: Thirty patients with CLD were studied and subjected to serum creatinine, total bilirubin, serum sodium, serum albumin, prothrombin time (PT), international normalized ratio (INR), esophagogastroduodenoscopy (upper gastrointestinal (UGI) endoscopy), and transjugular or transfemoral catheterization for HVPG measurement, and Child-Turcotte-Pugh (CTP) score and Model for End-Stage Liver Disease (MELD) score were calculated.

Results: The results indicates a strong positive connection between MELD and HVPG, which is statistically significant (r=0.754; p<0.001). Similarly, CTP and HVPG also exhibit a significant positive association (r=0.793; p<0.001) suggesting a link between the severity of liver disease. Additionally, the moderate positive correlation for encephalopathy has a significant value (r=0.584; p=0.001), while the weak positive correlations for serum bilirubin, INR, and HVPG have non-significant values (r=0.244; p=0.194, and r=0.375; p=0.041, respectively). A strong negative connection between serum albumin and HVPG was also found (r=0.546; p=0.005) suggesting a relationship between worsening liver function.

Conclusion: In patients with decompensated CLD, the severity of the CLD as measured by the CTP and MELD score corresponds with HVPG, and higher HVPG associated with severe CLD and severe ascites, large varices, and variceal hemorrhage. Higher HVPG in cirrhotic patients also suggests the existence of sequelae, such as varices, severe ascites, and severe hepatic encephalopathy, although HVPG has little bearing on the underlying cause.

## Introduction

Worldwide, chronic liver disease (CLD) may affect anybody, regardless of age, sex, location, or ethnicity. Cirrhosis is the outcome of several different liver disorders and is defined by architectural distortion of the liver and fibrosis, as well as the development of regenerative nodules. It also has many clinical symptoms and problems [1]. Cirrhosis is the leading source of morbidity and mortality on a global scale. In 2016, it caused 2.2% of global mortality and 1.5% of disability-adjusted life years, making it the 11th-leading cause of death and the 15th-leading cause of morbidity [2]. In 2017, 1.32 million individuals perished due to CLD, with approximately two-thirds of the deaths occurring in males and one-third in females [3]. In the Global Burden of Disease survey, the incidence of CLD and cirrhosis was recorded at 26 per 100,000 individuals in 2015. This rate exhibited a significant increase of 13% from the figures reported in the year 2000 [4]. Cirrhosis is projected to affect 26 out of every 100,000 people in Europe, while it affects 23.6 out of every 100,000 people in Southeast Asia [4].

Historically, viral hepatitis was the leading cause of CLD. However, enhanced hepatitis B and hepatitis C preventive measures and remedies have led to a decline in CLD trends. Globally, liver disease mortality rates have decreased over the past three decades, which reflects this trend. In particular, the age-adjusted mortality rate (AADR) for CLD decreased from 21 to 16.5 per 100,000 persons between 1990 and 2017 [5].

However, it's probable that CLD mortality rates are cautious and understate the full severity of the disease [6]. Alcohol-related liver disease (ALD), hepatitis B virus (HBV), non-alcoholic fatty liver disease (NAFLD), and chronic hepatitis C virus (HCV) are the most frequent etiologies of CLD and cirrhosis. The opioid crisis, obesity pandemic, and rising rates of alcohol abuse are just a few of the recent events that are changing the epidemiology of CLD and cirrhosis. Other recent changes include increased access to and efficacy of HCV therapy [7].

Data on CLD that are relevant to health policy are few in India [8]. Recent estimates, however, show that liver disease affected 1.28% of all hospitalized patients, with considerable geographical variations in the leading causes of CLD (hepatitis B in the East and South, hepatitis C in the North, NAFLD in the West, and ALD in the Northeast) [8].

An important complication and side effect of cirrhosis is portal hypertension (PHT). The primary clinical effects of cirrhosis are caused by PHT, a severe, almost inevitable complication of CLD. The most effective method presently available to assess the existence and severity of PHT is HVPG measurement. A rise in HVPG to 10 mmHg is considered clinically significant PHT; at this level, the consequences of PHT may begin to show. In clinical hepatology, measuring HVPG is becoming more common, and multiple studies have shown that the parameter is a reliable surrogate marker for challenging clinical end objectives. In the normal range, the portal vein pressure is 1-4 mmHg greater than the hepatic vein free pressure and never more than 6 mmHg higher than the right atrial pressure. PHT is defined by pressures higher than 10 mmHg [9]. It happens because of both increased portal venous blood flow and intrahepatic vascular resistance. Splanchnic arterial vasodilation increases portal blood flow, which in turn causes PHT to become more severe [10]. The direct measurement of portal pressure is a serious morbidity-producing invasive operation. A quick, risk-free approach that properly depicts the pressure of the portal vein in patients with cirrhosis (liver) is the assessment of the HVPG [11,12].

HVPG testing serves as the definitive method to assess the presence and severity of PHT in individuals with cirrhosis [13-15]. Even when an individual has an HVPG of 10 mmHg, which is considered clinically significant, they may experience mild and asymptomatic PHT, but it is still associated with an increased risk of cirrhosis-related complications such as ascites, encephalopathy, and variceal hemorrhage [16-18].

In the final stage of progressive liver fibrosis, the hepatic architecture is abnormal. In the early phases of cirrhosis, compensation occurs. The majority of people at this stage show no symptoms, and cirrhosis is commonly discovered incidentally during treatment for other illnesses. Consequently, the prevalence of compensated cirrhosis is often significantly underestimated in practical terms. When patients with compensated cirrhosis start to manifest symptoms such as ascites, esophageal variceal hemorrhage, hepatic encephalopathy (HE), and, in certain cases, an elevated bilirubin concentration, they are considered to be decompensating. Decompensation refers to the worsening of liver function in individuals with cirrhosis, and it often indicates a more advanced stage of the disease with the development of serious complications [19]. Since those experiencing decompensation are more likely to seek medical attention immediately, reports on the incidence of decompensated cirrhosis are likely to be significantly more accurate than those of compensated cirrhosis [20]. Depending on the cause of decompensation, the 1-year case-fatality rate for cirrhosis may reach as high as 80%. The incidence of morbidity and mortality caused by cirrhosis increases markedly after decompensation has occurred [21,22].

The level of liver fibrosis often corresponds with portal pressure, which is measured as the HVPG and may be used to predict the incidence of variceal bleeding. Additionally, a considerable drop in portal pressure after therapy may increase cirrhotic patients' long-term survival [23,24], implying that HVPG measurement may provide special information for result prediction. This research aimed to ascertain the relationship between the HVPG and its correlation with clinical and endoscopic parameters in CLD that have not yet been recompensated.

## Materials and methods

Study design 

The study was conducted in the Gastroenterology Department of Narayana Hospital-Rabindranath Tagore International Institute of Cardiac Sciences in Kolkata, India. The present study was a hospital-based observational study for a period of 12 months from August 2019 to July 2020. Patients who have allergic reactions, respiratory or psychiatric illness, severe cardiac disease, and hepatocellular carcinoma were excluded and patients diagnosed with decompensated CLD and aged more than 18 years who provided duly signed informed consent were included in the study.

Ethical approval

Prior to the commencement of the study, ethical clearance was obtained from the Institutional Ethics Committee of Narayana Hospital-Rabindranath Tagore International Institute of Cardiac Sciences in Kolkata, India, with ethical approval number: NHRTIICSEC/AP/011/2019.

Data collection

A total of 30 patients were interviewed to record their demographic details such as age, sex thorough medical history, and symptoms. For clinical data collection, patients were subjected to serum creatinine, total bilirubin, serum sodium, serum albumin, prothrombin time (PT), international normalized ratio (INR), esophagogastroduodenoscopy (upper gastrointestinal (UGI) endoscopy), and transjugular or transfemoral catheterization for HVPG measurement, and Child-Turcotte-Pugh (CTP) and Model for End-Stage Liver Disease (MELD) scores were calculated.

Procedure

HVPG Measurement

**Figure 1:**
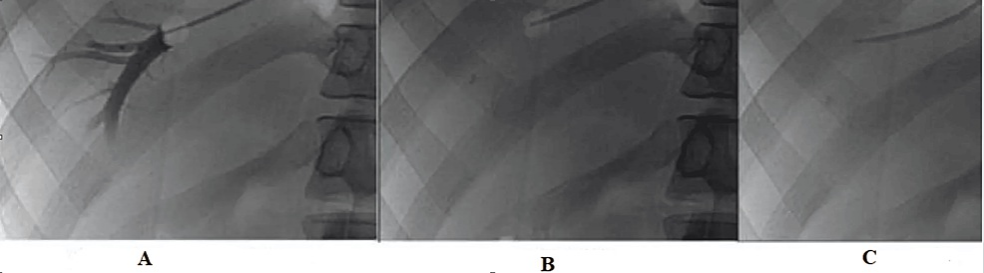
HVPG measurement. The HVPG (A) was then calculated by subtracting the FHVP (C) from the WHVP (B) HVPG: hepatic venous pressure gradient; FHVP: free hepatic venous pressure; WHVP: wedged hepatic venous pressure Image Credit: Dr. Amol Sathawane

The liver was catheterized using either the femoral vein or the right jugular vein. A double-lumen balloon-tipped Swan-Ganz catheter (Edwards Lifesciences, Irvine, California, United States) with a 7F or lower rating was inserted into the right or middle hepatic vein through the percutaneous transjugular or transfemoral route. The right or middle hepatic vein was entered 2 cm with the balloon catheter, and the free hepatic venous pressure (FHVP) was recorded. As the pressure was being continuously monitored, air was introduced into the balloon to wedge the hepatic vein to measure the wedged hepatic venous pressure (WHVP). By gently introducing 2 mL of contrast agent into the catheter to show the contrast agent's retention in the blocked portion of the hepatic vein, the wedged position was verified. The HVPG (A) was then calculated by subtracting the FHVP (C) from the WHVP (B) (Figure 1).

Varices

According to the standards of the American Association for the Study of Liver Diseases (AASLD), varices are classified as large (>5 millimeters) or small (≤5 millimeters) [25].

HE

The West Haven criteria were used to make the encephalopathy diagnosis [26]. Furthermore, patients were evaluated for the presence of variceal bleeding, ascites, CTP score, CTP class [27,28], MELD score [29], and etiology. All these findings were recorded on a predesigned and pretested proforma.

Statistical analysis

Quantitative data were compared using the Mann-Whitney test and reported as mean+standard deviation (SD). The χ2 test or Fisher's exact test was used to measure qualitative data. The IBM SPSS Statistics for Windows, Version 25.0 (Released 2017; IBM Corp., Armonk, New York, United States) was used to conduct statistical analysis. The mean HVPG levels were compared with ascites and varices using one-way analysis of variance (ANOVA). The cutoff for statistical significance was p≤0.05.

## Results

This one-year hospital-based observational study was conducted from August 2019 to July 2020 in the Department of Gastroenterology of Narayana Hospital-Rabindranath Tagore International Institute of Cardiac Sciences in Kolkata, India. A total of 30 patients with CLD were studied. The data obtained was analyzed and the results are depicted below.

Table 1 displays the demographic parameters of the sample. The research comprised a total of 30 participants with CLD where 20 (66.67%) were men and 10 (33.33%) were women. Patients were between the ages of 51 and 70. Ethanol was the major reason in etiology for causing CLD in 11 (36.68%) patients. Also, it was found that variceal bleeding was present in 23 (76.67%) and absent in seven (23.33%) patients. Severe and mild ascites were found in 18 and 10 (60 and 33.33%) patients, respectively, while it was absent in two (6.67%). Class B CTP was found in 10 (33.33%) and class C in 20 (66.67%) patients. Similarly, large and small varices were present in scale 20 (66.67%) and 8 (26.66%), respectively, while it was absent in two (6.67%) patients. HE was distributed in three grades where grade 1 was seen in 14 (46.67%), grade 2 in 13 (43.33%), and grade 3 in three (10%) patients.

**Table 1:** Demographic details of the patients HE: hepatic encephalopathy; CTP: Child-Turcotte-Pugh; PBC: primary biliary cholangitis

Parameters	No. of patients	Percentage
Gender		
Female	10	33.33
Male	20	66.67
Age (years)		
20-50	11	36.67
51-70	17	56.67
71-90	2	6.66
Etiology		
Ethanol	11	36.68
Cryptogenic	7	23.33
Hepatitis B	6	20
Hepatitis C	3	10
Autoimmune	1	3.33
PBC	1	3.33
Wilson disease	1	3.33
Variceal bleeding		
Present	23	76.67
Absent	7	23.33
Ascites		
Severe	18	60
Mild	10	33.33
Absent	2	6.67
CTP		
Class B	10	33.33
Class C	20	66.67
Varices		
Large	20	66.67
Small	8	26.66
Absent	2	6.67
HE grades		
Grade 1	14	46.67
Grade 2	13	43.33
Grade 3	3	10

The association of various parameters in the studied groups with HVPG is depicted in Table 2. The data indicated a highly significant association of HVPG with gastrointestinal bleeding, ascites, varices, and HE grades (Table 2).

**Table 2:** Association of parameters with HVPG HE: hepatic encephalopathy; HVPG: hepatic venous pressure gradient measurement; p-value<0.05, 0.01, 0.001 denotes statistical significance

Parameters		HVPG (mmHg) (mean±SD)	p-value
Gender	Male	16.55±4.68	0.973
	Female	16.60±3.27	
Gastrointestinal bleeding	Yes	17.86±3.86	<0.001
	No	12.28±1.70	
Ascites	Severe	19.11±2.67	<0.001
	Mild	13.40±2.87	
	Absent	9.50±0.70	
Varices	Large	19.00±2.63	<0.001
	Small	12.25±1.38	
	Absent	9.50±0.70	
HE grades	Grade 1	14.57±3.08	0.001
	Grade 2	17.15±3.89	
	Grade 3	23.33±2.30	
Ethanol	Yes	16.45±3.44	0.914
	No	16.63±4.68	

Also, both classes B and C of CTP indicated a highly significant association with HVPG (<0.001) (Table 3).

**Table 3:** Comparison of different parameters with HVPG CTP: Child-Turcotte-Pugh Score, p-value<0.05, 0.01, 0.001 denotes statistical significance

Parameters	Median	p-value
CTP		
Class B	12.00	<0.001
Class C	19.00	

Correlation between CTP, MELD, encephalopathy, serum bilirubin, serum albumin, and INR

**Figure 2:**
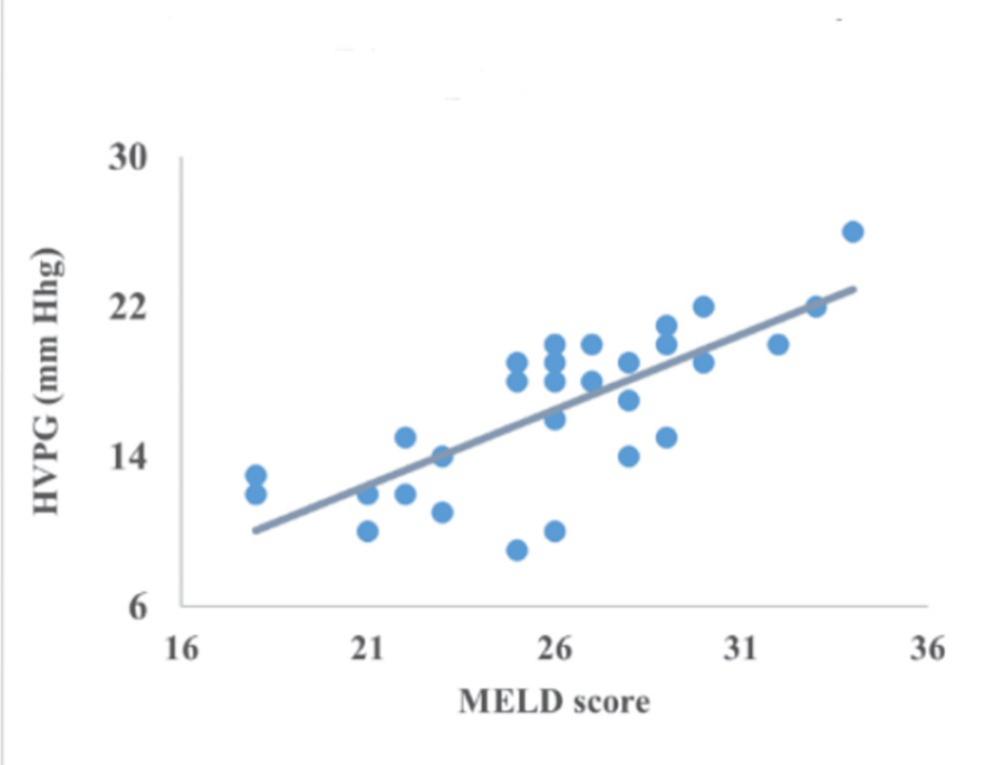
Correlation between MELD score and HVPG HVPG: hepatic venous pressure gradient; MELD: Model for End-Stage Liver Disease Correlation coefficient=0.784; p-value=0.001 Image Credit: Dr. Harshal Khobragade

**Figure 3:**
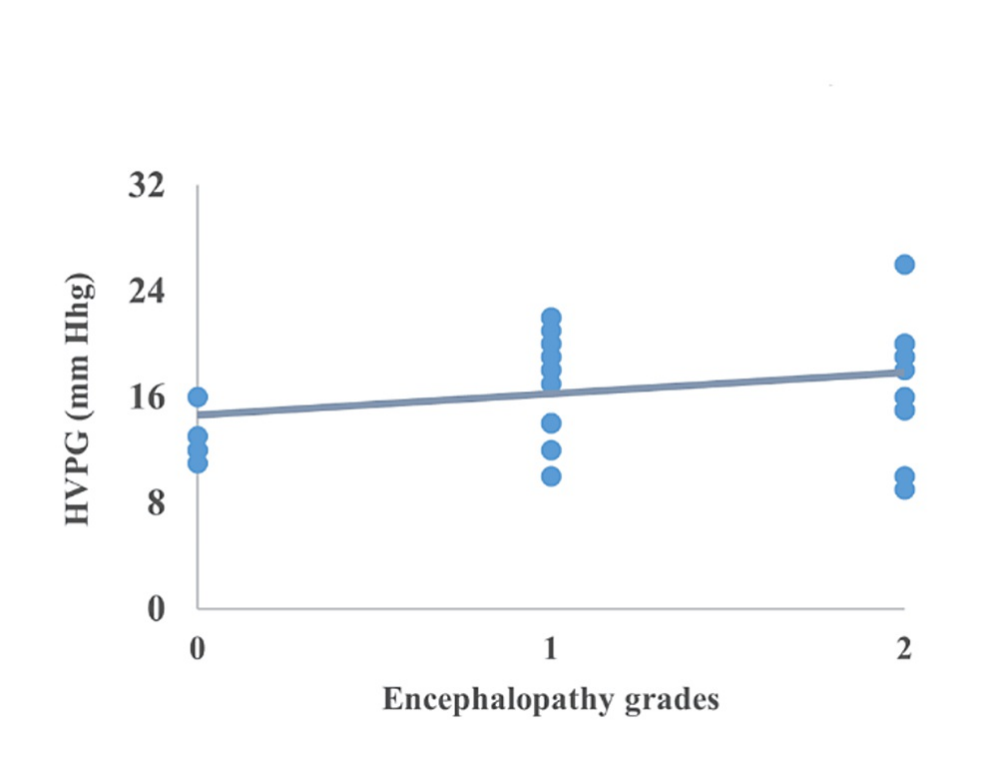
Correlation between encephalopathy grades and HVPG HVPG: hepatic venous pressure gradient Correlation coefficient=0.584; p-value=0.001 Image Credit: Dr. Harshal Khobragade

**Figure 4:**
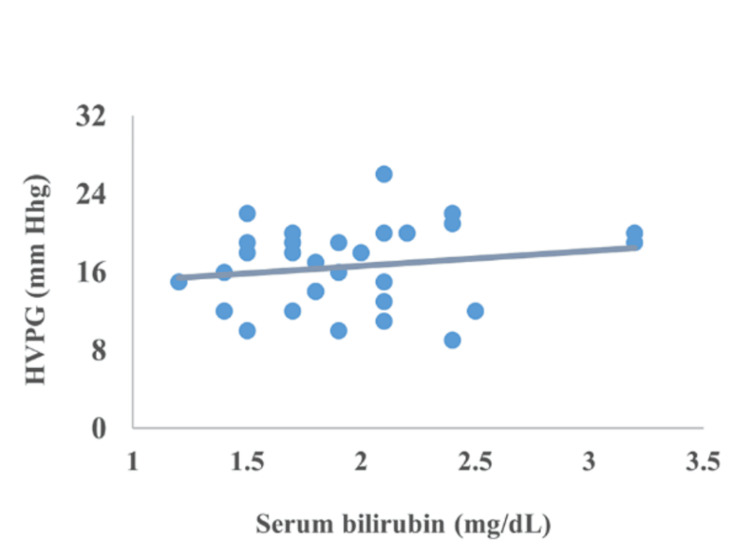
Correlation between serum bilirubin and HVPG HVPG: hepatic venous pressure gradient Correlation coefficient=0.244; p-value=0.194 Image Credit: Dr. Harshal Khobragade

**Figure 5:**
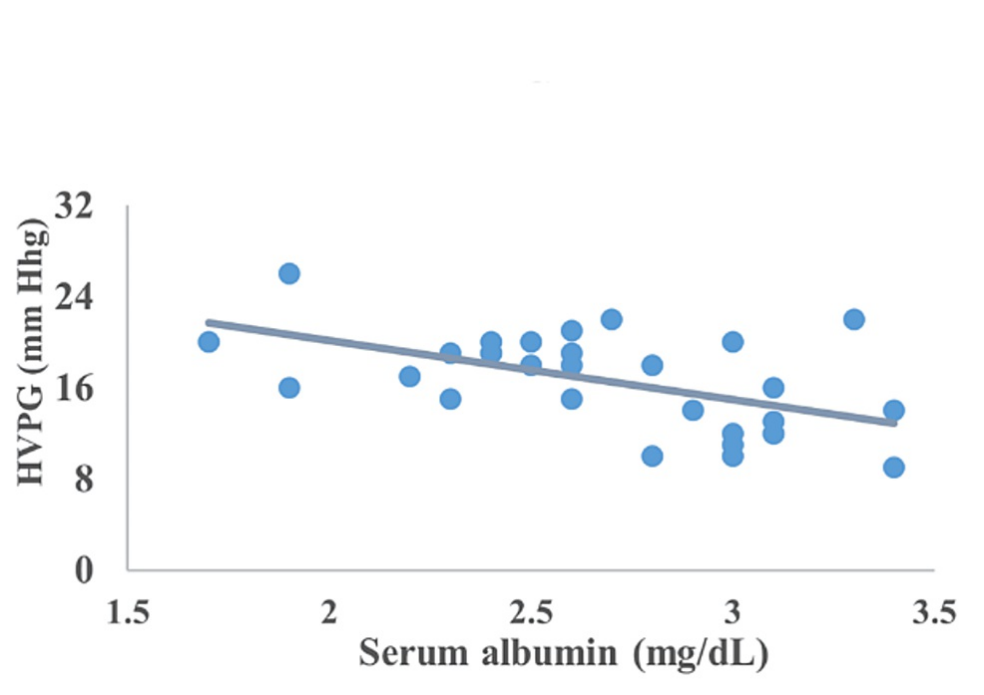
Correlation between serum albumin and HVPG HVPG: hepatic venous pressure gradient Correlation coefficient=0.546; p-value=0.005 Image Credit: Dr. Harshal Khobragade

**Figure 6:**
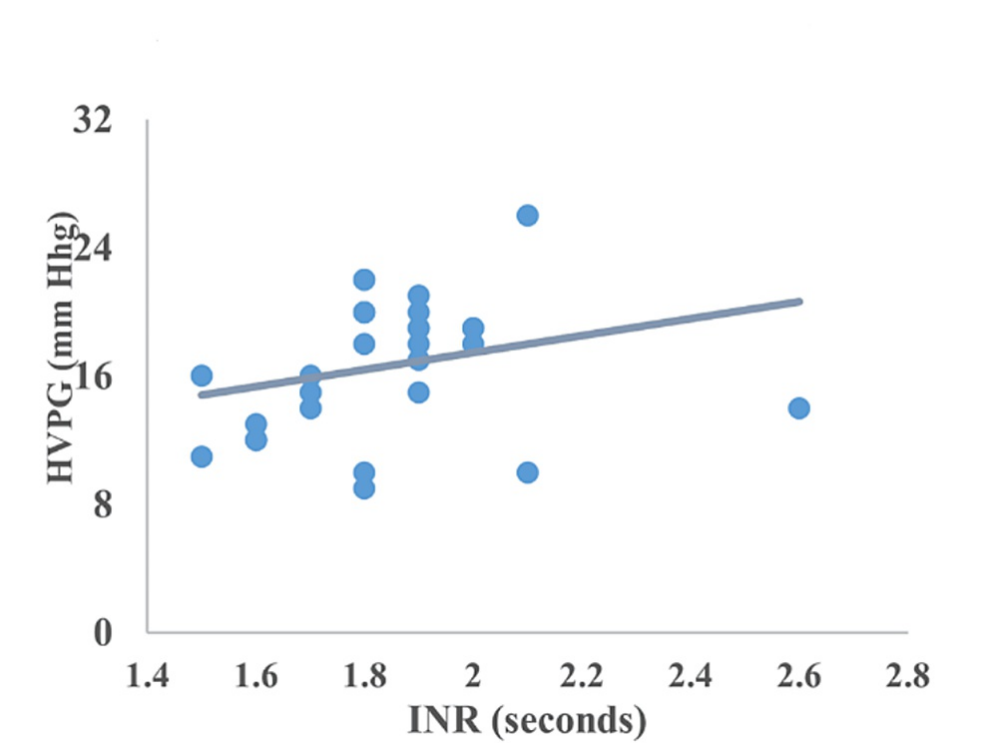
Correlation between INR and HVPG HVPG: hepatic venous pressure gradient; INR: international normalized ratio Correlation coefficient=0.375; p-value=0.041 Image Credit: Dr. Harshal Khobragade

**Figure 7:**
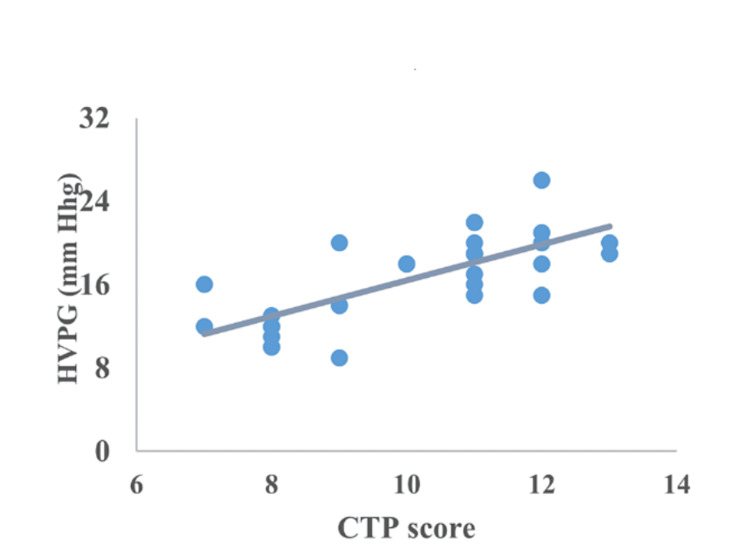
Correlation between CTP score and HVPG HVPG: hepatic venous pressure gradient; CTP: Child-Turcotte-Pugh Correlation coefficient=0.754; p-value=0.001 Image Credit: Dr. Harshal Khobragade

The correlation of HVPG with CTP, MELD, encephalopathy, serum bilirubin, serum albumin, and INR is represented in Figures 2-7. The figures represent a very significant positive correlation (r=0.754, p=0.001) between CTP, MELD, and HVPG. Additionally, a weak positive insignificant correlation was discovered between serum bilirubin and HVPG (r=0.244; p=0.194), and a weak positive significant correlation was discovered between HVPG and INR (r=0.375; p=0.041), while a significant moderate positive correlation was observed between HVPG and encephalopathy (r=0.584; p=0.001). Serum albumin and HVPG also showed a negative connection (r=0.546; p=0.005) (Figures 2-7).

## Discussion

CLD is characterized by constant intrahepatic vascular remodeling that includes sinusoidal capillarization, fibrogenesis, and neoangiogenesis. Liver failure is a negative effect of this increased hepatic resistance, which also raises portal pressure and decreases the efficiency of hepatocyte blood circulation. When managed properly, HVPG measurement is a reliable and predictive tool for managing PHT [30].

An increasing body of research suggests that HVPG may be able to predict the risk of variceal hemorrhage as well as overall liver-related outcomes. Higher HVPG might indicate a greater chance of decompensation and worse survival rates. The most popular predictive techniques are the MELD and CTP evaluations. In considering these elements, the present study sought to assess the relationship between HVPG, CTP, and MELD scores, variceal bleeding, ascites, variceal size, and the etiology of liver cirrhosis in patients.

In the present study, 66.67% of the patients were males and 33.33% were females and the male-to-female ratio was 2:1 suggesting the occurrence of liver disease twice in males than females. Further, the mean HVPG in males and females were statistically similar (16.55±4.68 vs 16.60±3.27 mmHg; p=0.973). The sex distribution pattern noted in the present study was similar to the observations reported by Ramanathan et al. where they reported a male-to-female ratio as high as 5:1 [31]. Similar results were seen by Mukherjee et al. [8] concluding that 73% of the males had CLD. In this study, ages ranged from 23 to 81 years with more than one-third of the patients (40%) aged from 51 to 60 years suggesting that the occurrence of CLD was common in the middle-aged group. The findings were strongly supported by the findings of Silkauskaite et al. [32] and Kim et al. [33].

When compared to patients with class B CTP in this study, individuals with class C CTP had considerably higher median HVPG. The observations suggested that there is a strong correlation between CTP and HVPG indicating that the higher the HVPG, the severe the CLD. These observations were consistent with the previous reports by Lee et al. [34]. 

The severity of liver disease and the likelihood of death are both correlated with the patient's MELD score. It has also been linked to a higher incidence of transplantation without increasing mortality among those on the transplant waiting list. Although it has not been conclusively shown, the MELD score and HVPG level are good indicators of the real severity of liver disease. Researchers Liu et al. [35] and Kim et al. [33] discovered a strong and positive association between the MELD score and HVPG level, which was supported by the results of the present study.

According to the findings of the current study, individuals with large varices exhibited significantly higher mean HVPG levels compared to those with small varices or without varices. These results align with a study conducted by Al Mahtab et al. [36] in India, where patients with varices demonstrated a notably elevated mean HVPG level (11.7±3.8) compared to those without varices. The statistical analysis revealed a negligible significance of varices when comparing smaller (16.2±4.9 mmHg) and larger (20±7 mmHg) sizes.

The leading cause of morbidity and death in cirrhotic patients is bleeding from esophageal varices. Cirrhotic individuals are more likely to develop esophageal varices than the general population, with a lifetime frequency of around 80-90%. The findings of our research indicate variceal bleeding in most of the patients (76.67%) corresponding with the results of Gulzar et al. [37], in which bleeding was seen in a significant proportion of patients. Higher HVPG levels may indicate variceal bleeding in cirrhotic individuals because in the current investigation, the mean HVPG in patients with variceal bleeding was considerably greater than that in patients without variceal hemorrhage. 

In the present study, ethanol was the etiology of CLD in more than one-third of the patients (36.68%). The findings of the Lithuanian research by Silkauskaite et al. [32] also found ethanol as the cause of CLD in more than one-third of the patients with liver disease.

The similarity between alcoholic and non-alcoholic CLD suggests that individuals with CLD have elevated HVPG levels despite the underlying etiology. It needs further verification using a sample size that is appropriate to the reason. Although there was no correlation between the mean HVPG and etiology in the current study, the mean HVPG was marginally higher in alcoholic etiologies than in viral and other etiologies, according to Ramanathan et al. from India [31]. The findings showed statistically non-significant results, suggesting that individuals with viral etiology cirrhosis might benefit from the existing consensus on treating PHT, which is mostly based on research involving persons with alcoholic cirrhosis.

Patients with HE grade 3 had a significantly greater HVPG diagnosis rate compared to those with HE grades 2 and 1. There was a moderately significant positive connection between HE grades and HVPG. Since encephalopathy patients were excluded from the studies by Al Mahtab et al. [36], Kim et al. [33], and Silkauskaite et al. [32], other studies have investigated the correlation between HE and HVPG. In contrast to the findings of the current study, Ramanathan et al. observed that there was no link between HE and HVPG and that there was not a significant distinction between those with mild encephalopathy and those without encephalopathy in terms of the mean HVPG level [31].

Serum albumin and HVPG in the current research had a substantial, moderately negative association, whereas INR and HVPG had a significant, weakly positive correlation. These findings were in line with prior research from India by Ramanathan et al. [31].

Overall, the present study revealed that the severity of the CLD as ascertained by CTP and MELD score correlates with HVPG in patients with cirrhosis and higher HVPG prompts severe CLD and severe ascites, large varices, and variceal bleeding. At the same time, higher HVPG in patients with cirrhosis also hints the presence of complications, that is, varices, severe ascites, and severe HE, but HVPG has limited significance to etiology. 

The study's limitation lies in its relatively smaller sample size. Additionally, due to the smaller subset of patients with diverse etiologies, conducting subgroup analyses, such as etiology- and gender-specific analyses, was not feasible. Lastly, the study did not encompass a consideration of long-term outcomes, as it fell beyond the scope of this investigation.

## Conclusions

The HVPG test is a reliable, safe, and easy way to detect liver fibrosis. The severity of the CLD as determined by CTP and MELD score correlates with HVPG in patients with decompensated CLD, and higher HVPG prompts severe CLD and severe ascites, large varices, and variceal bleeding. At the same time, higher HVPG in patients with cirrhosis also hints the presence of complications, that is, varices, severe ascites, and severe HE, but HVPG has limited significance to etiology. The measurement of HVPG and the monitoring of the liver fibrosis stage are vital aspects of addressing CLD. Therefore, evaluating the cost-effectiveness of HVPG assessment becomes pivotal in CLD treatment, alongside observing hemodynamic consequences and staging liver fibrosis. 

## References

[1] Fiel MI (2010). Pathology of chronic hepatitis B and chronic hepatitis C. Clin Liver Dis.

[2] (2020). Global Health Estimates. https://www.who.int/data/global-health-estimates.

[3] (2020). The global, regional, and national burden of cirrhosis by cause in 195 countries and territories, 1990-2017: a systematic analysis for the Global Burden of Disease Study 2017. Lancet Gastroenterol Hepatol.

[4] Wong MC, Huang JL, George J (2019). The changing epidemiology of liver diseases in the Asia-Pacific region. Nat Rev Gastroenterol Hepatol.

[5] Mokdad AA, Lopez AD, Shahraz S (2014). Liver cirrhosis mortality in 187 countries between 1980 and 2010: a systematic analysis. BMC Med.

[6] Asrani SK, Larson JJ, Yawn B, Therneau TM, Kim WR (2013). Underestimation of liver-related mortality in the United States. Gastroenterology.

[7] Moon AM, Singal AG, Tapper EB (2020). Contemporary epidemiology of chronic liver disease and cirrhosis. Clin Gastroenterol Hepatol.

[8] Mukherjee PS, Vishnubhatla S, Amarapurkar DN (2017). Etiology and mode of presentation of chronic liver diseases in India: a multi centric study. PLoS One.

[9] Abraldes JG, Sarlieve P, Tandon P (2014). Measurement of portal pressure. Clin Liver Dis.

[10] Menon KV, Kamath PS (2001). Regional and systemic hemodynamic disturbances in cirrhosis. Clin Liver Dis.

[11] Bellis L, Castellacci R, Montagnese F, Festuccia F, Corvisieri P, Puoti C (2003). Hepatic venous pressure gradient determination in patients with hepatitis C virus-related and alcoholic cirrhosis. Eur J Gastroenterol Hepatol.

[12] Perelló A, Escorsell A, Bru C, Gilabert R, Moitinho E, García-Pagán JC, Bosch J (1999). Wedged hepatic venous pressure adequately reflects portal pressure in hepatitis C virus-related cirrhosis. Hepatology.

[13] Groszmann RJ, Glickman M, Blei AT, Storer E, Conn HO (1979). Wedged and free hepatic venous pressure measured with a balloon catheter. Gastroenterology.

[14] Bosch J, Mastai R, Kravetz D, Bruix J, Rigau J, Rodés J (1985). Measurement of azygos venous blood flow in the evaluation of portal hypertension in patients with cirrhosis. Clinical and haemodynamic correlations in 100 patients. J Hepatol.

[15] Bosch J, Mastai R, Kravetz D, Navasa M, Rodés J (1986). Hemodynamic evaluation of the patient with portal hypertension. Semin Liver Dis.

[16] Blasco A, Forns X, Carrión JA (2006). Hepatic venous pressure gradient identifies patients at risk of severe hepatitis C recurrence after liver transplantation. Hepatology.

[17] Garcia-Tsao G, Groszmann RJ, Fisher RL, Conn HO, Atterbury CE, Glickman M (1985). Portal pressure, presence of gastroesophageal varices and variceal bleeding. Hepatology.

[18] Ripoll C, Groszmann R, Garcia-Tsao G (2007). Hepatic venous pressure gradient predicts clinical decompensation in patients with compensated cirrhosis. Gastroenterology.

[19] Moreau R, Jalan R, Gines P (2013). Acute-on-chronic liver failure is a distinct syndrome that develops in patients with acute decompensation of cirrhosis. Gastroenterology.

[20] Fleming KM, Aithal GP, Card TR, West J (2010). The rate of decompensation and clinical progression of disease in people with cirrhosis: a cohort study. Aliment Pharmacol Ther.

[21] D'Amico G, Garcia-Tsao G, Pagliaro L (2006). Natural history and prognostic indicators of survival in cirrhosis: a systematic review of 118 studies. J Hepatol.

[22] Fleming KM, Aithal GP, Card TR, West J (2012). All-cause mortality in people with cirrhosis compared with the general population: a population-based cohort study. Liver Int.

[23] Merkel C, Bolognesi M, Sacerdoti D, Bombonato G, Bellini B, Bighin R, Gatta A (2000). The hemodynamic response to medical treatment of portal hypertension as a predictor of clinical effectiveness in the primary prophylaxis of variceal bleeding in cirrhosis. Hepatology.

[24] Abraldes JG, Tarantino I, Turnes J, Garcia-Pagan JC, Rodés J, Bosch J (2003). Hemodynamic response to pharmacological treatment of portal hypertension and long-term prognosis of cirrhosis. Hepatology.

[25] Garcia-Tsao G, Sanyal AJ, Grace ND, Carey WD (2007). Prevention and management of gastroesophageal varices and variceal hemorrhage in cirrhosis. Am J Gastroenterol.

[26] Ferenci P, Lockwood A, Mullen K, Tarter R, Weissenborn K, Blei AT (2002). Hepatic encephalopathy--definition, nomenclature, diagnosis, and quantification: final report of the working party at the 11th World Congresses of Gastroenterology, Vienna, 1998. Hepatology.

[27] Turcotte JG, Child CG 3rd (1967). Portal hypertension. Pathogenesis, management and prognosis. Postgrad Med.

[28] Malinchoc M, Kamath PS, Gordon FD, Peine CJ, Rank J, ter Borg PC (2000). A model to predict poor survival in patients undergoing transjugular intrahepatic portosystemic shunts. Hepatology.

[29] Kamath PS, Wiesner RH, Malinchoc M (2001). A model to predict survival in patients with end-stage liver disease. Hepatology.

[30] Thalheimer U, Bellis L, Puoti C, Burroughs AK (2011). Should we routinely measure portal pressure in patients with cirrhosis, using hepatic venous pressure gradient (HVPG) as a guide for prophylaxis and therapy of bleeding and rebleeding? No. Eur J Intern Med.

[31] Ramanathan S, Khandelwal N, Kalra N, Bhatia A, Dhiman RK, Duseja AK, Chawla YK (2016). Correlation of HVPG level with ctp score, MELD score, ascites, size of varices, and etiology in cirrhotic patients. Saudi J Gastroenterol.

[32] Silkauskaite V, Pranculis A, Mitraite D, Jonaitis L, Petrenkiene V, Kupcinskas L (2009). Hepatic venous pressure gradient measurement in patients with liver cirrhosis: a correlation with disease severity and variceal bleeding. Medicina (Kaunas).

[33] Kim TY, Suk KT, Jeong SW (2019). The new cutoff value of the hepatic venous pressure gradient on predicting long-term survival in cirrhotic patients. J Korean Med Sci.

[34] Lee JG, Sohn JH, Jeong JY, Kim TY, Kim SM, Cho YS, Kim Y (2020). Combined effect of hepatic venous pressure gradient and liver stiffness on long-term mortality in patients with cirrhosis. Korean J Intern Med.

[35] Liu C, Shao R, Wang S (2020). The presence of ascites affects the predictive value of HVPG on early rebleeding in patients with cirrhosis. Gastroenterol Res Pract.

[36] Al Mahtab M, M Noor E Alam S, A Rahim M (2017). Hepatic venous pressure gradient measurement in Bangladeshi cirrhotic patients: a correlation with child's status, variceal size, and bleeding. Euroasian J Hepatogastroenterol.

[37] Gulzar GM, Zargar SA, Jalal S (2009). Correlation of hepatic venous pressure gradient with variceal bleeding, size of esophageal varices, etiology, ascites and degree of liver dysfunction in cirrhosis of liver. Indian J Gastroenterol.

